# Pancytopenia during tyrosine kinase inhibitor treatment – coexistence of chronic myeloid leukemia and visceral leishmaniasis: a case report

**DOI:** 10.1186/s13256-016-0978-4

**Published:** 2016-07-27

**Authors:** Acy Quixada, Pedro Aurio Maia Filho, Tarcísio Paulo Almeida Filho, Fernando Barroso Duarte, Caroline Aquino Moreira-Nunes, Romélia Pinheiro Gonçalves Lemes

**Affiliations:** 1 Department of Haematology, Walter Cantidio University Hospital, 1210 Captain Francisco Pedro St, 60430-350, Rodolfo Teofilo, Fortaleza, Ceará Brazil; 2 Research Laboratory in Hemoglobinopathies and Genetics of Hematologic Diseases, Federal University of Ceará, Fortaleza, Ceará Brazil; 3 Department of Surgery, Federal University of Ceará, Fortaleza, Ceará Brazil

**Keywords:** Chronic myeloid leukemia, Tyrosine kinase inhibitors, Visceral leishmaniasis

## Abstract

**Background:**

Visceral leishmaniasis is a zoonosis characterized by chronic evolution of symptoms; it usually appears 2 to 4 months after the initial infection, with multiple cutaneous lesions and systemic involvement, which if left untreated results in death in 90 % of cases.

**Case presentation:**

We present a case of 29-year-old white male farmer, with chronic myeloid leukemia treated with imatinib who developed significant pancytopenia, leading to discontinuation of treatment. His neutrophil count fell to 0.5 × 10^9^/L, his platelets dropped to 85 × 10^9^/μL, and his hemoglobin was 6.4 g/dL. A bone marrow study was performed, showing complete remission of chronic myeloid leukemia and numerous *Leishmania* amastigotes within the macrophages. He used pentavalent antimonials replaced by amphotericin B due to acute cardiac toxicity. After 3 months, imatinib was restarted, and he again showed adequate control of the disease. The last polymerase chain reaction assessment showed a deep molecular response.

**Conclusion:**

The hypothesis of an adverse event or secondary resistance to tyrosine kinase inhibitors, with subsequent progression to advanced disease, was initially raised, although a detailed evaluation has shown that it was an associated infectious disease.

## Background

Chronic myeloid leukemia (CML) is a clonal myeloproliferative disorder that results from the malignant transformation of a hematopoietic stem cell. It is characterized by the Philadelphia chromosome (Ph+), which is formed by translocation and fusion of the long arms of chromosomes 9 and 22 in a multipotent hematopoietic progenitor cell. At the molecular level, the fusion generates a BCR-ABL protein with constitutive tyrosine kinase activity [1–7]. Tyrosine kinase inhibitors (TKIs) such as imatinib are able to suppress the BCR-ABL+ clone and induce molecular remission [8].

Leishmaniasis is a zoonosis caused by an intracellular parasite belonging to the genus *Leishmania. Leishmania* parasites have a dimorphic life cycle, alternating between an extracellular promastigote and an intracellular amastigote form [9]. This disease is endemic in 98 countries, and an estimated 350 million people live in endemic areas. Despite T-cell-dependent immune responses, which produce asymptomatic and self-healing infection, or appropriate treatment, intracellular infection is probably lifelong because the targeted cells (tissue macrophages) allow residual parasites to persist [10].

## Case presentation

A 29-year-old white male farmer sought medical attention because of left upper-quadrant abdominal discomfort and unintentional weight loss of 8 kg. His initial clinical examination revealed light pallor and splenomegaly, 10 cm below his left costal margin. However, no lymphadenopathy or hepatomegaly was detected. Laboratory analysis showed a leukocyte count of 38 × 10^9^/L, a hemoglobin level of 10.5 g/dL, and a platelet count of 289 × 10^9^/μL. He denied fever or drenching night sweats and was not taking medication.

He was referred to our Department of Hematology, at our university hospital, where a bone marrow aspiration and biopsy were performed. Microscopic examination showed a left-shifted granulopoiesis, and the Ph + was found with no other chromosomic aberrations. BCR-ABL fusion transcript was identified by polymerase chain reaction (PCR) and both transcripts (b2a2 and b3a2) were present. The diagnosis of CML in chronic phase was made: Sokal score 0.85 (low risk <0.8; moderate risk 0.8 to 1.2; high risk >1.2) and Hasford score 741 (low risk <780; moderate risk 780 to 1480; high risk >1480).

He was initially treated with hydroxyurea, which was later substituted by imatinib 400 mg daily starting in early January 2013. We observed a slow progression of the splenomegaly until complete spleen regression by 3 months. The first reevaluation was made at 3 months of imatinib (early response). During that period he showed no symptoms and gained weight (4 kg). Early response was detected through a cytogenetics analysis and quantitative polymerase chain reaction (qPCR) assessment. There was no cell growth to perform an analysis of the Ph + through the G-band method. Fluorescent *in situ* hybridization revealed 3.5 % of cells with the gene fusion, while PCR assessment was not conclusive due to the low quality of deoxyribonucleic acid (DNA). He was considered an optimal responder.

**Fig. 1:**
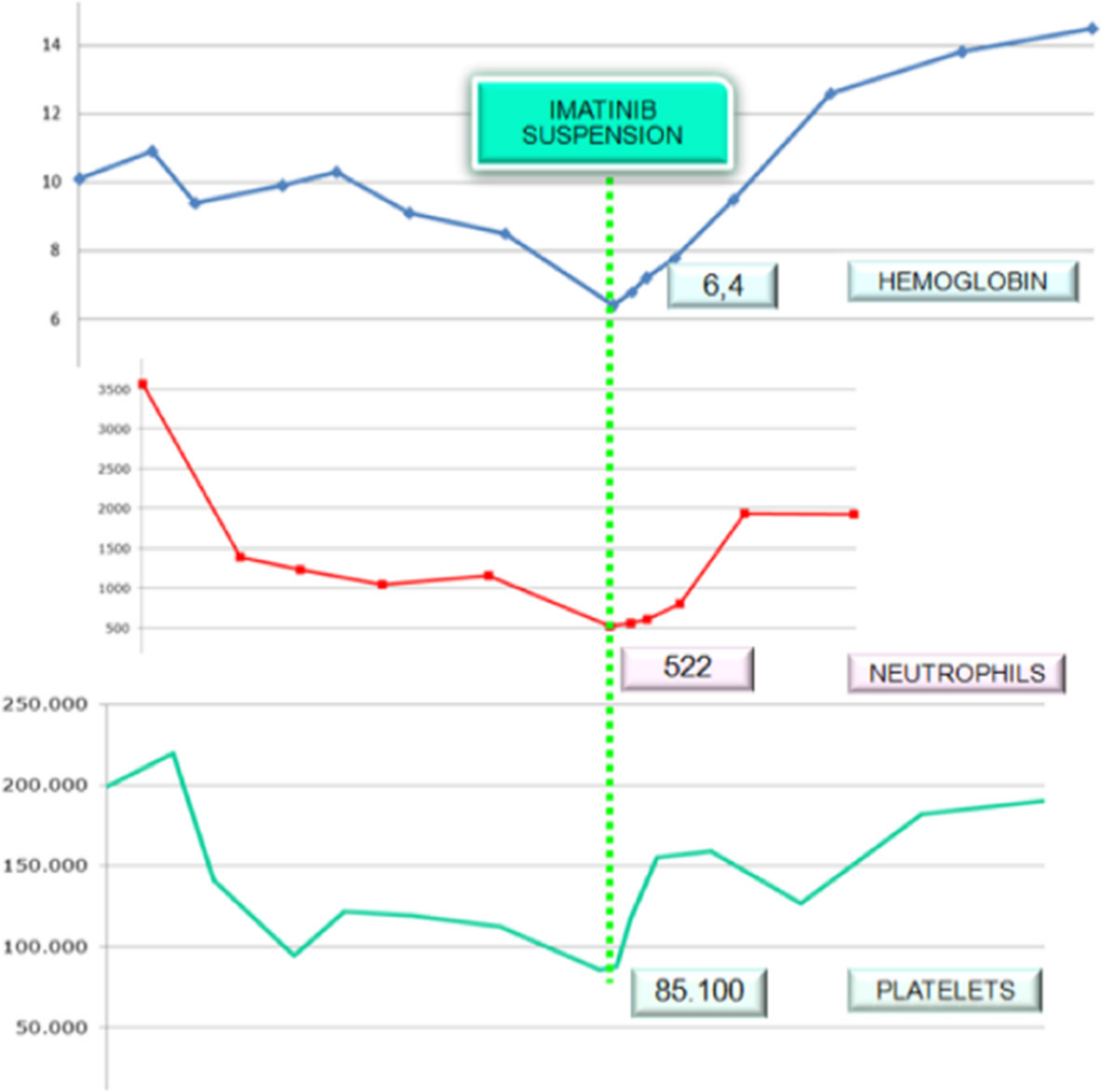
Evolution of patient’s hemogram. The *dotted line* indicates the nadir counts (when imatinib suspension occurred)

**Fig. 2:**
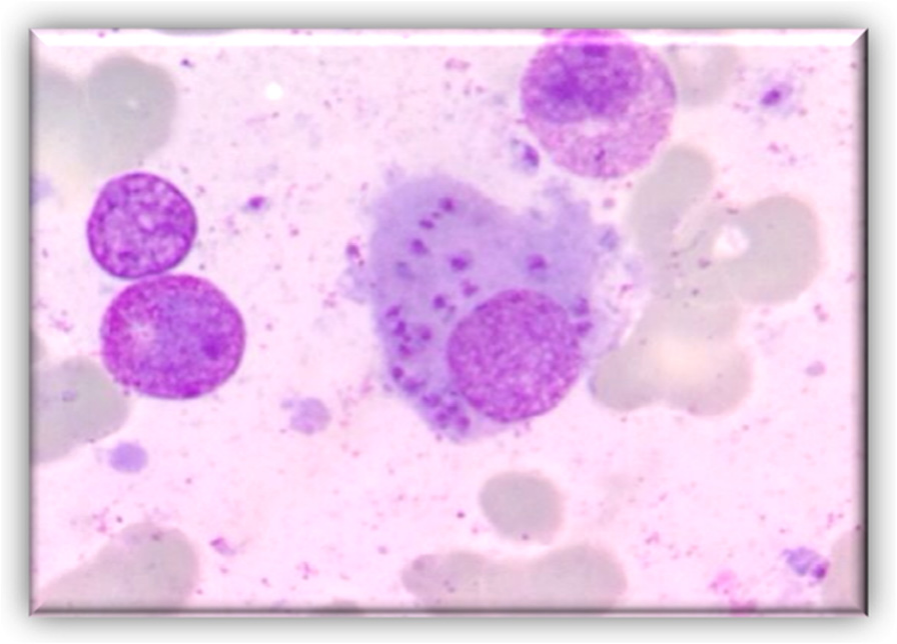
Bone marrow aspirate: macrophages containing Leishman-Donovan bodies

**Fig. 3:**
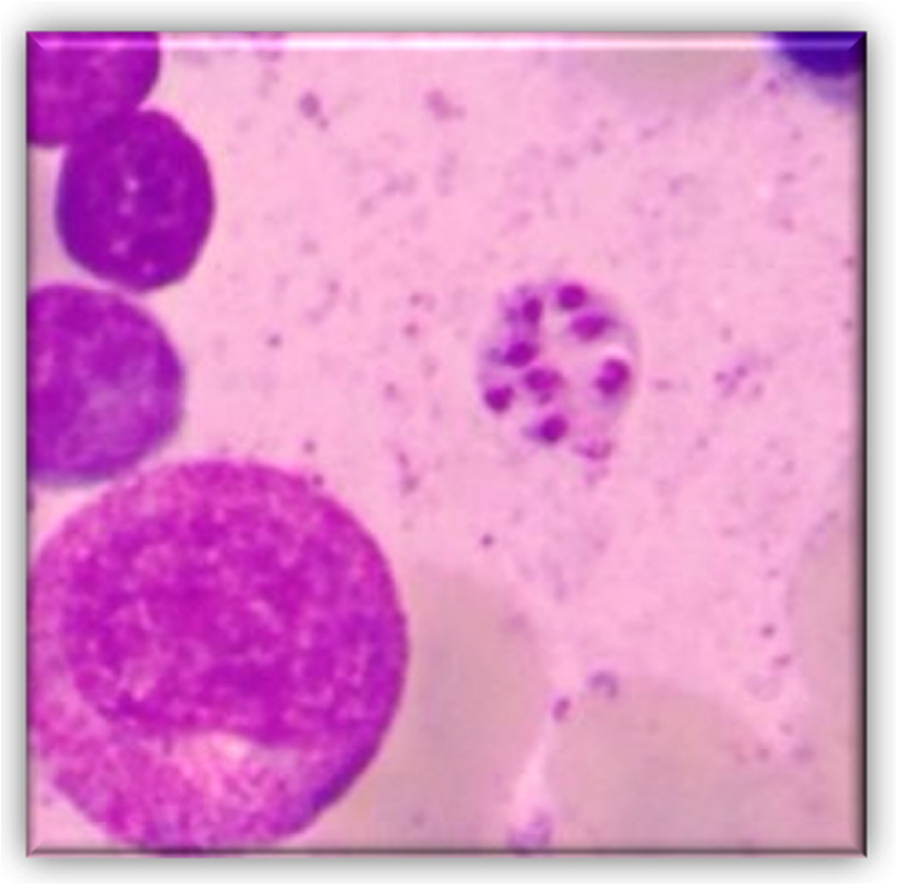
Bone marrow aspirate: Leishman-Donovan bodies outside macrophages

He persisted on imatinib 400 mg for 6 months before the agent was discontinued because of pancytopenia. His neutrophil count fell to 0.5 × 10^9^/L, platelets dropped to 85 × 10^9^/μL, and hemoglobin was 6.4 g/dL (Fig. 1). Transfusion was not necessary. After imatinib cessation, we waited 1 week until a new hemogram was done. Because there was no improvement in his hemogram we decided to examine his bone marrow. A bone marrow study was performed showing complete remission of CML and numerous *Leishmania* amastigotes within the macrophages (Figs. 2 and 3). IgM antibody to *Leishmania* K39 antigen was positive. He was admitted to our hospital and began treatment with pentavalent antimonials. His hemogram started to improve at 10 days of leishmaniasis treatment and normalized by 2 months. New bone marrow aspirate showed no more leishmaniasis after 60 days of therapy. Complete recovery of symptoms and examinations (bone marrow and hemogram) was seen after that time (Table 1).

**Table 1:** Clinical and laboratory findings in the patient during leishmaniasis diagnosis and therapy

	Before	During treatment	After
Hemoglobin (g/dL)	6.4	9.5	12.6
Neutrophil (×10^9^/L)	0.5	1.7	2
Platelet (×10^9^/μL)	85	150	145

Acute cardiac toxicity required suspension from treatment. An electrocardiogram showed prolongation of the QT interval and bradycardia. The medication was stopped when his heart rate dropped below 45 beats per minute; it was replaced by amphotericin B regimen. After 3 months, TKI therapy was resumed and he again showed adequate response to imatinib. The last PCR showed deep molecular response (MR 4.0).

## Discussion

Visceral leishmaniasis (VL) is a well-known opportunistic infection in severe immunodeficiency, particularly in patients living in areas where leishmaniasis is endemic. In Brazil, VL or neotropical kala-azar, is an endemic/epidemic rural and periurban anthropozoonosis with a strong tendency towards urbanization. It is caused by different species of the genus *Leishmania*, and in the Americas, *L. chagasi* is the etiological agent of the disease. The importance of the canine reservoir derives from the close frequent contact between dogs and humans and the fact that the animals can present asymptomatic infection, despite a high degree of parasitism in healthy skin and viscera [11, 12].

We report the case of a farmer diagnosed with CML who presented with pancytopenia Grade 3/4 during treatment, which required imatinib suspension. Adherence was good, without the use of any other drugs. Profound involvement of the hematological system in the form of bone marrow and peripheral blood changes are consistently seen in VL and include the following.

Normochromic normocytic anemia is a frequent and clinically significant feature of VL and hemoglobin levels of 7 to 10 g/dl are commonly found. Leucopenia is an early and striking manifestation of VL. Platelet counts are usually affected after long duration of illness. A varying degree of frequency and severity of pancytopenia has been reported. In this case we think that both imatinib treatment and concurrent *Leishmania* infection played a role in the cytopenias observed.

The most commonly reported Grade 3 or 4 adverse events experienced in 5 years of follow-up of newly diagnosed patients with CML treated with imatinib as a first-line therapy were neutropenia, thrombocytopenia, anemia, and elevated liver enzymes [13]. In our patient, the diagnosis of leishmaniasis was not anticipated. Pancytopenia was considered an adverse event, and spleen enlargement after imatinib cessation was attributed to TKI secondary resistance/progression. Adverse hematologic side-effects of imatinib include anemia, neutropenia, and thrombocytopenia. Hematologic toxicity appears mild to moderate in most instances and is often easily manageable and potentially reversible. Less than 10 % of the patients present persistent cytopenias that might preclude treatment. By the time of imatinib cessation our concern was about pancytopenia as an adverse effect. When we stopped imatinib we observed a progressive spleen enlargement. Our patient had low grade fever and was a farmer. We then arrived at a second diagnostic hypothesis. When a new evaluation of his bone marrow was performed, the infectious disease was diagnosed.

## Conclusions

To the best of our knowledge, this report highlights the importance of careful management of patients with CML during treatment, with the formulation of different hypotheses on common situations in an attempt to increase the chance of elucidation of the etiologic diagnosis. Social context must always be taken into account. We should always try to explain the clinical conditions considering a single disease, but we need to recognize the possibility of a second pathology.

## Abbreviations

CML, chronic myeloid leukemia; PCR, polymerase chain reaction; Ph+, Philadelphia chromosome; qPCR, quantitative polymerase chain reaction; TKI, tyrosine kinase inhibitor; VL, visceral leishmaniasis
